# Synergy between surfactants’ stiffness and concentration on their self-assembly into reverse micelles as water droplet carriers in nonpolar solvents

**DOI:** 10.1371/journal.pone.0294913

**Published:** 2024-02-02

**Authors:** J.D. Hernández Velázquez, A. Gama Goicochea

**Affiliations:** Tecnológico Nacional de México, Tecnológico de Estudios Superiores de Ecatepec, División de Ingeniería Química y Bioquímica, Estado de México, Mexico; State University of New York at Binghamton: Binghamton University, UNITED STATES

## Abstract

A study of the self-assembly process into reverse micelles (RMs) of linear surfactants and monomeric aqueous solutes dissolved in nonpolar solvents, varying the concentration (*c*_*s*_) and the persistence length (*L*_*p*_) of the surfactants is presented here. The influence of *c*_*s*_ and *L*_*p*_ on the structural and dynamic properties of the aggregates is investigated through mesoscopic simulations carried out with the dissipative particle dynamics method. All simulations are performed at a fixed water/surfactant molecular ratio of 2:1, varying the surfactant concentration from *c* = 6 *wt*% up to *c* = 12 *wt*%, for increasing surfactants’ rigidity from *L*_*p*_ = 0.73 nm up to *L*_*p*_ = 44.99 nm. It is found that there exists a collaborative interplay between *c*_*s*_ and *L*_*p*_ that enhances the number of RMs assembled and their diffusion as carriers of water droplets. These results should be useful as guidelines to understand and improve processes where the RMs are implemented to carry aqueous solutes in nonpolar solvents.

## 1. Introduction

Reverse micelles (RMs) are self-organized agglomerations formed by amphiphilic surfactants when these are dissolved in nonpolar or organic solvents. Although there is evidence indicating that the RMs formation is influenced by the addition of water [1, 2] or polar solutes [3, 4], the self-assembly of surfactants into stable RMs in nonpolar solvents also occurs in the absence of polar solutes [5, 6]. RMs have a wide range of technological applications, such as in the synthesis of nanomaterials [7–9], catalytic processes [10, 11], and drug delivery systems [12, 13]. They are important also in different industrial processes, such as in the food industry as protein extractors [14, 15], and as thickeners of supercritical carbon dioxide (scCO_2_) for the enhanced oil recovery and fracking industries [16–19].

Within the scope of numerical simulations, the atomistic study of reverse micellar systems has provided useful insights about the mechanisms behind the self-assembly process at molecular- and atomistic-levels [20–25]. Earlier molecular dynamics (MD) simulations carried out by Brown and Clarke [21], provided information about the structural properties of a single insolated RM as a water droplet carrier in a nonpolar solvent. On the other hand, studies of the self-assembly process forming RMs in specific nonpolar solvents arose a decade after. For instance, Tobias and Klein [26] were the first to investigate the local properties of RMs formed by a mixture of water/calcium carbonate/calcium sulfonate, in the nonpolar carbon tetrachloride and octane solvents. Salaniwal and collaborators [22] were the first to study the self-assembly process of water/surfactant [(C_7_F_15_)(C_7_H_15_)CHSO_4_^-^Na^+^)] aggregates in scCO_2_. Allen *et al*. [23] studied the local properties of RMs formed by water/poly(oxyethylene) surfactants in decane as solvent.

The importance of coarse-grained numerical methods relies on their capacity to simulate systems at larger length and time scales in comparison with atomistic-level techniques, such as MD. Dissipative particle dynamics (DPD) is a coarse-grained simulation method developed to study the hydrodynamic properties of complex fluids [27, 28]. Several DPD works have delved into the study of the mechanisms that lead to the self-assembly of different classes of surfactants into direct [29, 30] or reverse micelles [31–33]. Recently, Lavagnini *et al*. [34] provide a detailed report about the parametrization of the chemical structure of various types of ester-, amide-, and sugar-based surfactants into DPD-parametrized structures. They modeled the self-assembly of 27 different surfactants in aqueous solvents and found that the critical micelle concentration predicted by their simulation agreed with the available experimental data [34]. The work carried out by Lin and co-workers [35] directly implemented a RM-based system for the extraction of proteins, using the DPD method. They proved that results obtained for the recovery efficiency of the protein were validated with experimental data found in the literature [35].

Despite all the work there is around RMs, it is still necessary to invest efforts in the study of these systems to improve the understanding of both the self-association process in the formation of aggregates and the technological/industrial implementation of RMs. This work presents the study of structural and dynamic properties of the self-assembly process of linear surfactants into RMs in nonpolar solvents [33]. It has been reported that the self-assembly of linear surfactants into RMs is modulated by their persistence length (*L*_*p*_), for fixed molecular water/surfactant ratio of 1:1 and at constant surfactant’s concentration (*c*_*s*_) [33]. Here, cooperative effects are reported on the structural and dynamic properties of the self-assembly process forming water/surfactant aggregates in nonpolar solvents. Our results show that all RMs have roughly spherical shape regardless of the concentration or the surfactant’s rigidity. However, the structure of the surfactant’s tail groups that envelopes the water/surfactant headgroups’ nuclei depends on the rigidity degree of the surfactants. It is also found that the number of RMs and their diffusion depend on the competition/cooperation between the surfactants’ concentration and their persistence length.

## 2. Models and methods

### (a) Dissipative particle dynamics method

All simulations were carried out using the coarse-grained DPD simulation method [27, 36]. The DPD model is similar to the standard MD simulation algorithm, in the sense that the equation of motion of all the particles making up the system is solved simultaneously to obtain their position and momenta [37]. The DPD force-field consists of three pairwise, additive central forces, namely the conservative ($F_{ij}^{C}$), dissipative ($F_{ij}^{D}$) and the random forces ($F_{ij}^{R}$) [36]. The latter two forces are given by

$$F_{ij}^{D}=-\gamma \omega^{D}\left(r_{ij}\right)\left(r_{ij}\cdot v_{ij}\right){\hat{r}}_{ij},$$ (1)

$$F_{ij}^{R}=\sigma \omega^{R}\left(r_{ij}\right)\xi_{ij}{\hat{r}}_{ij},$$ (2)

where **r**_*ij*_ and **v**_*ij*_ are the relative position and velocity vectors between the *i*-th and *j*-th particles, respectively; and ${\hat{r}}_{ij}=r_{ij}/|r_{ij}|$ is the unit position vector. The symbols *ξ*_*ij*_ = *ξ*_*ji*_ are random numbers with Gaussian properties. The weight functions $\omega^{D}\left(r_{ij}\right)={\left[\omega^{R}\left(r_{ij}\right)\right]}^{2}=1-r_{ij}/r_{c}^{*}$ make of these short-range forces that drop to zero when the relative distance between the *i*-th and *j*-th particles (*r*_*ij*_) is larger than the cutoff radius ($r_{c}^{*}$). The parameters *γ* and *σ* are the maximum strength of the dissipative and random forces, respectively, which are coupled through the fluctuation-dissipation theorem. The balance between these forces yields a built-in thermostat, such that *k*_*B*_*T* = σ^2^/2*γ* [36]. Here, *k*_*B*_ is Boltzmann’s constant and *T* is the absolute temperature.

The remaining of the three fundamental DPD forces is the conservative force ($F_{ij}^{C}$), defined as a soft, linearly decaying repulsive force that also drops to zero when $r_{ij}\geq r_{c}^{*}$:

$$F_{ij}^{C}=a_{ij}\left(1-r_{ij}/r_{c}^{*}\right){\hat{r}}_{ij}.$$ (3)

In Eq (3), *a*_*ij*_ is the maximum force intensity between the *i*-th and *j*-th particles. Table 1 shows the values of the parameters *a*_*ij*_ used in this work, which have been successfully tested in previous reports. In particular, with the parameters in Table 1, faster aggregation was achieved of both direct (using Gemini surfactants) [38] and reverse micelles (using linear surfactants) [33] in aqueous and nonpolar solvents, respectively. Further details for the calculation of *a*_*ij*_ parameters can be found elsewhere [39].

**Table 1:** Values of the DPD conservative force parameters, *a*_*ij*_ (see Eq (3)), for each pair of DPD particle species. All values are in reduced DPD units (see section 2 (b)). Fig 1(A) shows the schematic representation of all DPD particle species.

	Water (W)	Oil (O)	Head (H)	Tail (T)
**Water (W)**	78.3	140.0	50.0	140.0
**Oil (O)**		78.3	140.0	78.3
**Head (H)**			90.0	78.3
**Tail (T)**				78.3

**Fig 1:**
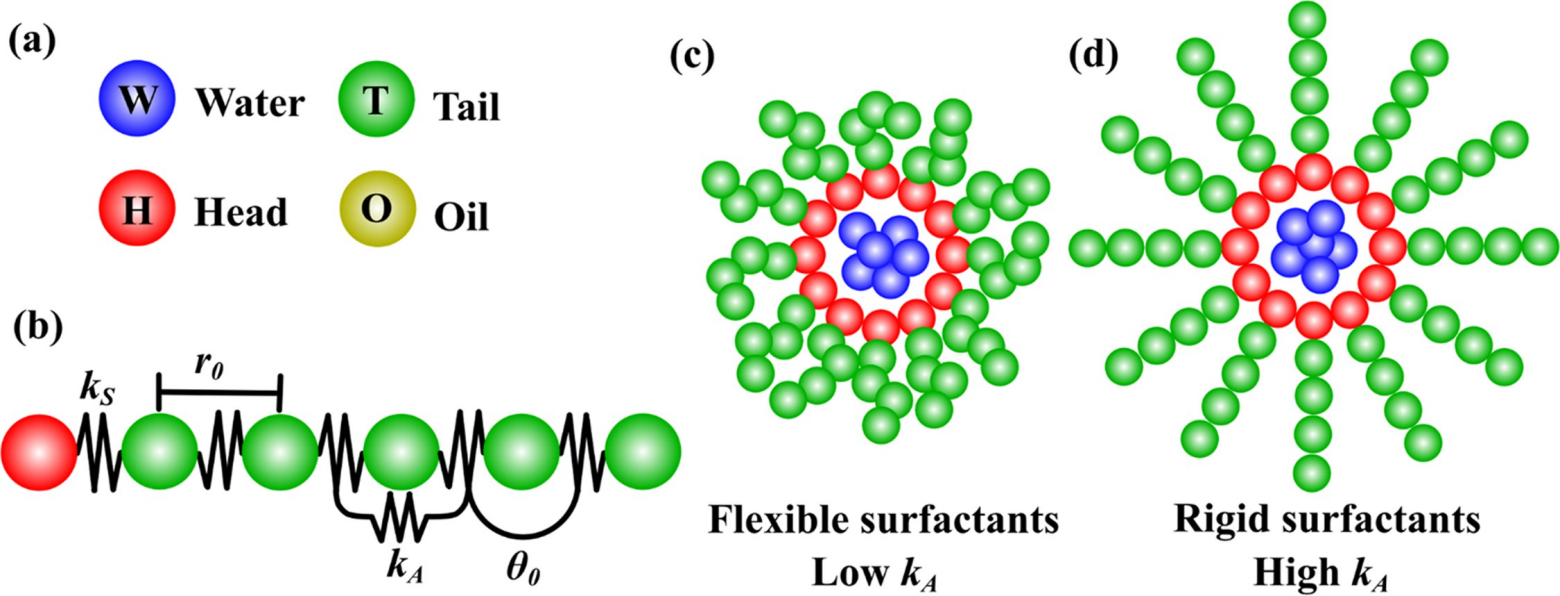
(a) DPD species used in all simulations, water (W, in blue), surfactant’s head (H, in red), surfactant’s tail (T, in green), and oil (O, in dark yellow). (b) HT5 surfactant model. The bonding and bending forces’ parameters used to join consecutive beads of the surfactants are schematically represented. The constants *k*_*S*_ and *k*_*A*_ are the spring and angular constants of $F_{ij}^{S}$ (Eq (4)) and $F_{ij}^{A}$ (Eq (5)), respectively. The equilibrium distance and angle are *r*_0_ and *θ*_0_; see $F_{ij}^{S}$ (Eq (4)) and $F_{ij}^{A}$ (Eq (5)), respectively. In (c) and (d), one finds simplified representations of the aggregates formed by flexible and rigid surfactants, respectively.

To model chain-like, (H)ead-(T)ail linear structures, such as the HT5 surfactant, with one DPD head bead and 5 beads in the tail used in this work, two harmonic, conservative forces are added to the model. One of them models the bonding forces between neighboring surfactant beads. The Kremer-Grest bead-spring model [40] is used to join two consecutive beads along the surfactant, using a harmonic oscillator:

$$F_{ij}^{S}=-k_{S}\left(r_{ij}-r_{0}\right){\hat{r}}_{ij},$$ (4)

where *k*_*S*_ and *r*_0_ are the spring constant and its equilibrium distance, respectively. The second harmonic force is introduced to model the rigidity of each surfactant chain, which is an angular spring based on a three-body force, given as follows:

$$F_{ijk}^{A}=-k_{A}\sin\left(\theta_{ijk}-\theta_{0}\right),$$ (5)

where *θ*_*ijk*_ is the angle formed by three consecutive beads in the surfactant. In Eq (5), *k*_*A*_ and *θ*_0_ are the angular spring constant and the equilibrium angle, respectively. Fig 1(B) shows a simplified representation of the two bonding forces (Eqs (4) and (5)) used to model the HT5 surfactant. Fig 1(C) and 1(D) illustrate the consequences of having a RM made up of flexible and rigid surfactants, respectively. Eqs (1)–(5) make up the force field used here to study the ternary mixture systems of oil/water/surfactant, in the water/surfactant self-assembly process into RMs.

### (b) Simulation details

All quantities used in the simulations are expressed in reduced DPD units, which are listed in Table 2.

**Table 2:** Reduced DPD units and their conversion factors. The SI conversion factors correspond to the scenario when the coarse-grained degree is *N*_*m*_ = 3, which means that each DPD particle represents a group of three water molecules at room temperature [39, 41]. The asterisks refer to reduced DPD units.

DPD units		SI conversion factors
Mass [m^*^]	m^*^ = 1*m*	*m* = *N*_*m*_ (3×10^-23^g)
Length $\left[r_{c}^{*}\right]$	$r_{c}^{*}$ = 1 *r*_*c*_	*r*_*c*_ = 6.46 × 10^−10^ m
Time [Δ*t*]	Δ*t* = 0.03 τ	τ = 3.012 × 10^−12^ *s*
Energy [E^*^]	E^*^ = 1 *k*_*B*_*T*	*k*_*B*_*T* = 4.14 × 10^−21^ J

All simulations were performed under canonical ensemble conditions, where the number density and the global temperature of the systems are always fixed. All systems studied here were placed in a cubic simulation of $L_{x}^{*}=L_{y}^{*}=L_{z}^{*}=17.1r_{c}^{*},$ using periodic boundary conditions in all the faces of the box. The total number of DPD particles in all systems was fixed at *N* = 1.5 × 10^4^, to keep a constant reduced number density of ρ^*^ = 3. The reduced temperature was always kept equal to one [36]. All simulations reported here were carried out in two stages. In the first, systems were run for over 30 blocks of 10^4^ time steps (Δ*t*) each, to allow the system to reach thermal equilibrium. In the production stage, the systems were run for an additional 20 blocks of 10^4^Δ*t* each, where most of the properties of interest are averaged. Considering the two stages, the total simulation time of each system was ∼45.18 *ns*.

The parameters of the Kremer-Grest bead-spring force (Eq (4)) of each HT5 surfactant were chosen as $k_{S}=100k_{B}T^{*}/r_{c}^{*2}$ and $r_{0}=0.7r_{c}^{*}$ [33]. These parameters have been successfully used to model different types of linear-chain structures [33, 42], demonstrating that are appropriate to prevent bond crossing [43]. The interplay between surfactant concentration and persistence length on the water/surfactant self-assembly into RMs, was studied for four surfactant concentrations (*c*_*s*_) and five values of the persistence length (*L*_*p*_). Here, the value of the angle of equilibrium was set in *θ*_0_ = 180° to model a linear structure, while the persistence length is related to the angular spring constant *k*_*A*_ (see Eq (5)) as follows [44]:

$$L_{p}=-b_{0}/\text{ln}\left[L\left(k_{A}\right)\right].$$ (6)

In Eq (6), *b*_0_ is the average bond length, such that $b_{0}=1/\sqrt[{3}]{\rho^{*}}\approx 0.7r_{c}^{*}$; whereas *L*(*k*_*A*_) = coth(*k*_*A*_)-(1/*k*_*A*_) is the Langevin function [44]. Table 3 shows the different surfactant concentrations and persistence lengths modeled in this work.

**Table 3:** Values of the surfactant concentration (*c*_*s*_) and the surfactant persistence length (*L*_*p*_) studied in this work. The values of the persistence length were obtained using Eq (6) for the different values of *k*_*A*_ shown in this table.

***c***_***s***_ [***wt*** %]	6	8	10	12	
** $k_{A}\left[E^{*}/r_{c}^{*}\right]$ **	2	5	10	20	100
***L***_***p***_ [**nm**]	0.73	2.03	4.29	8.82	44.99

## 3. Results

To understand the role played by the concentration of linear surfactants and their rigidity on their self-assembly into RMs, a set of simulations of water/HT5 surfactant mixtures at fixed 2:1 molecular ratio was carried out. The surfactant concentration was changed proportionally to the total weight percentage of the system, considering four different surfactant concentrations, *c*_*s*_. The rigidity of the HT5 surfactant was given by its persistence length, *L*_*p*_ (Table 3 lists the values of *c*_*s*_ and *L*_*p*_ studied in this work). Thus, the cooperative effects of *c*_*s*_ and *L*_*p*_ on the structural and dynamic properties of the water/HT5 aggregates formed in nonpolar solvents, were studied from a mesoscopic point of view.

**Fig 2:**
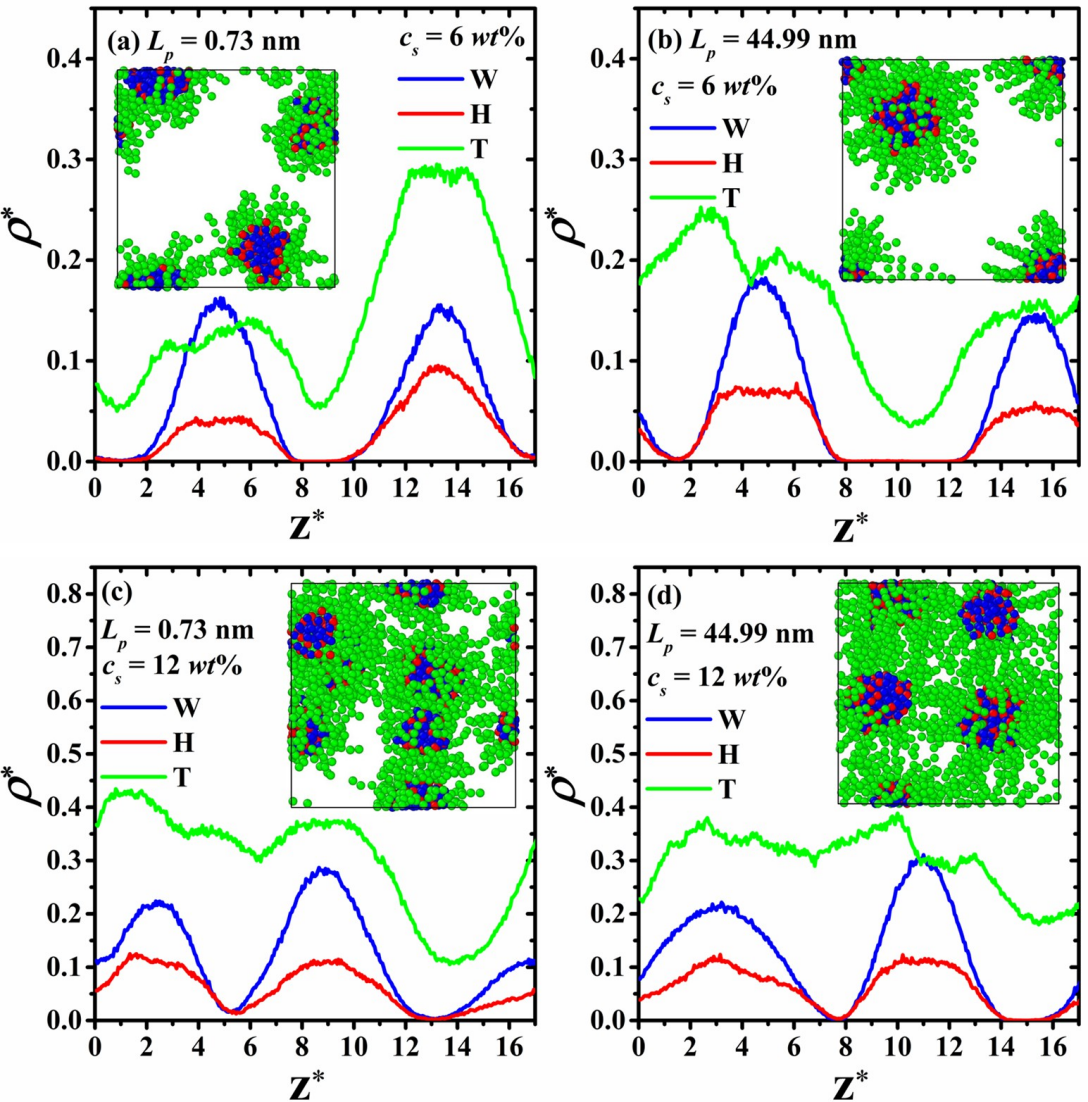
Density profiles of water beads (W, blue lines), surfactant’s head (H, red lines), and surfactant’s tail (T, green lines) beads, from the last simulation block of the systems. The left-hand panels ((a) and (c)) show the density profiles of surfactants with the lowest persistence length, while in the right-hand panels ((b) and (d)) display the density profiles of surfactants with the highest persistence length. The density profiles of the nonpolar solvent are omitted for clarity.

Let us start with the structural analysis of the density profiles of the systems shown in Fig 2, comparing the effects produced by the stiffness of the HT5 on the micellization process at the lowest (*c*_*s*_ = 6 *wt*%, Fig 2(A) and 2(B)) and highest surfactant concentrations (*c*_*s*_ = 12 *wt*%, Fig 2(C) and 2(D)). Fig 2 shows the reduced number density profiles along the *z*-direction of the simulation box, omitting the nonpolar solvent profiles, for visualization purposes. All profiles displayed in Fig 2 correspond to the last simulation block of the production phase. The rest of the density profiles are found in S1 Fig in the S1 File accompanying this article.

As expected, the self-assembly process yields the formation of RMs, and the water beads dissolved in the nonpolar solvent are encapsulated in the RMs. It is to be noted that in all cases, all the water beads dissolved in the system are captured inside the micelles’ nuclei, as seen in the snapshots in each panel of Fig 2. The blue and red particles correspond to the water beads and surfactants’ headgroups, respectively. This encapsulation can also be observed in the density profiles, since the water beads’ profiles (blue lines in Fig 2(A)–2(D)) and surfactants’ heads profiles (red lines in Fig 2(A)–2(D)) follow each other closely. The maxima in the water beads and surfactants’ heads profiles are located at the same *z*-position, although they differ in intensity, as a consequence of the water/surfactant molecular ratio of 2:1. Additionally, one can note that the micelle’s nuclei are always covered by surfactants’ tail groups, as seen in their density profiles. This is shown by the green lines in Fig 2(A)–2(D)), which always surround the maxima of the components forming the micelle’s nuclei, i.e., water beads and surfactants’ heads (blue and red lines in Fig 2(A)–2(D), respectively).

Recently, it has been demonstrated that the number of RMs formed by linear surfactants models, such as the one used in this work, can be measured from their density profiles by counting the number of maxima of water beads or surfactants’ heads (components of the RMs nuclei) [33]. However, in the surfactant concentration range studied here (from *c*_*s*_ = 6 *wt*% up to *c*_*s*_ = 12 *wt*%), it becomes more difficult to identify how many aggregates each maximum corresponds to. For instance, in the case of the most rigid surfactants (*L*_*p*_ = 44.99 nm) at *c*_*s*_ = 6 *wt*%, two RMs are formed due to the water/HT5 aggregation (see snapshot in Fig 2(B)). This also agrees with the number of maxima of the water beads’ (and surfactants’ heads) density profile displayed in Fig 2(B). As *c*_*s*_ increases, see for example Fig 2(D), it becomes increasingly difficult to associate the number of RMs formed to maxima observed in the density profile of the water beads or that of the surfactants’ heads. Nevertheless, the water beads are always encapsulated by the surfactants’ heads, as shown in all panels in Fig 2.

**Fig 3:**
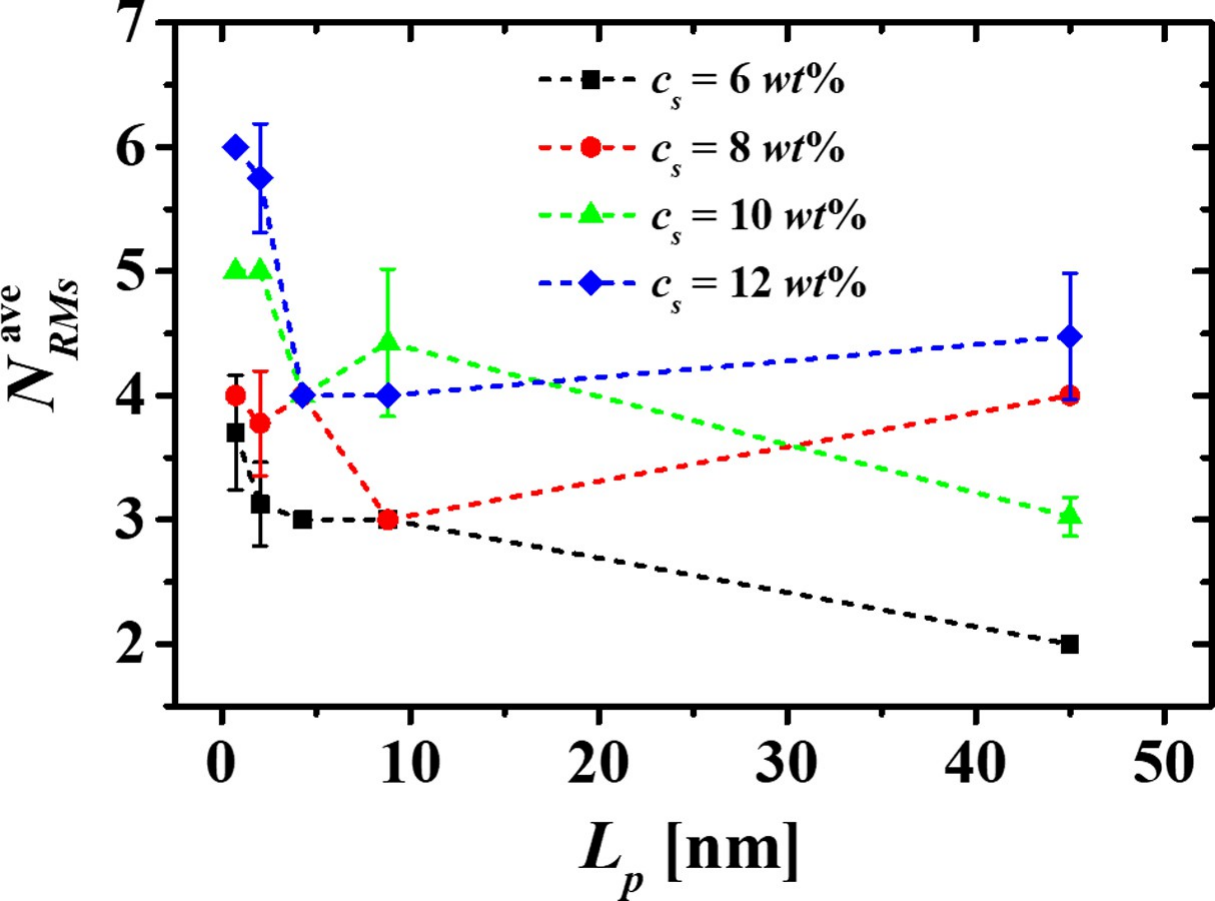
Average number of RMs, $N_{RMs}^{ave}$, as a function of the surfactant persistence length (*L*_*p*_) for the four different surfactant concentrations. The averages were calculated over 18.072 ns, and the number of RMs was counted every 451.8 ps.

Different simulation studies have demonstrated that the number of RMs formed by linear surfactants in nonpolar solvents depends on the system’s conditions. Among those are the water/surfactant molecular ratio and the type of solvent in which the micellization process occurs [2]. The number of RMs depends also on some physical properties of the surfactants, such as their stiffness and tail group’s length [33]. Our results show that by increasing *c*_*s*_ at fixed water/HT5 molecular ratio of 2:1, higher number of RMs are found, which encapsulate all water beads dissolved in the nonpolar medium, regardless of the stiffness of the HT5. On the other hand, fewer RMs are formed when increasing *L*_*p*_ for a fixed surfactant concentration in most cases, as seen in Fig 3, where the average number of RMs ($N_{RMs}^{\text{ave}}$) is plotted as a function of *L*_*p*_ for the four different concentrations.

As Fig 2 shows, the HT5 surfactant model used in this work exhibits perfect efficiency as water-droplet container. In all the systems studied, either at different surfactant concentrations or for the different surfactant’s persistence length, all the water beads dissolved were encapsulated by the RMs, see also S1 Fig in the S1 File. Furthermore, it is found that the self-assembly process leading to the RMs formation is stable as a function of time, since the number of RMs formed did not change throughout the course of the simulations. This is in contrast to the case of Gemini surfactants, whose self-association process leads to the formation of unstable micelles, with dynamic aggregation/segregation process for different types of architectures such as Π-shaped [45] or X-shaped [38]. The aggregation mechanism leading to the formation of RMs reported here for the HT5 surfactant is stable enough that if two RMs fuse into a single RM, no water beads inside the nuclei are released during the aggregation process.

**Fig 4:**
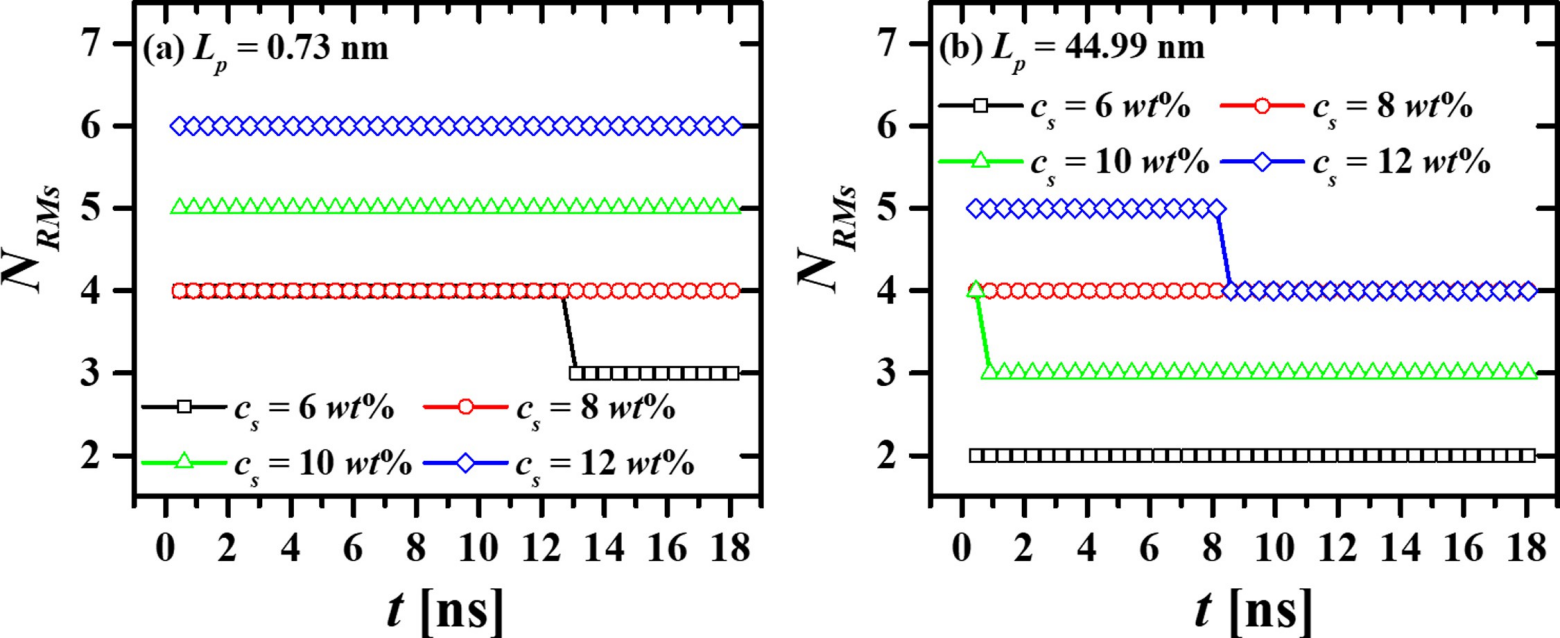
Time evolution of the number of RMs (*N*_*RMs*_) formed during the self-assembly process, as a function of time. Here, only results for the systems made up of the HT5 surfactants with the lowest (a) and highest rigidity (b) are shown, for clarity. The number of RMs was counted every 451.8 ps for a span of 18.072 ns.

To demonstrate the stability of the micellization process, the time evolution of the number of RMs (*N*_*RMs*_) formed by the most flexible (Fig 4(A)) and the most rigid surfactants (Fig 4(B)), was tracked for all the surfactant concentrations studied. Fig 4 shows that, once the RMs are assembled, they do not break up. The results corresponding to the cases with *L*_*p*_ = 2.03 nm, 4.89 nm, and 8.82 nm, are found in S2 Fig in the S1 File. These results also reveal that there exists synergy between *L*_*p*_ and *c*_*s*_, leading to enhanced self-assembly between the water beads and the HT5 surfactant. The number of aggregates formed during the micellization process depends on *L*_*p*_ and *c*_*s*_; thus, the more rigid the surfactants are, the lower the number of aggregates is. On the other hand, the number of aggregates tends to grow with surfactant concentration, regardless of their rigidity.

**Fig 5:**
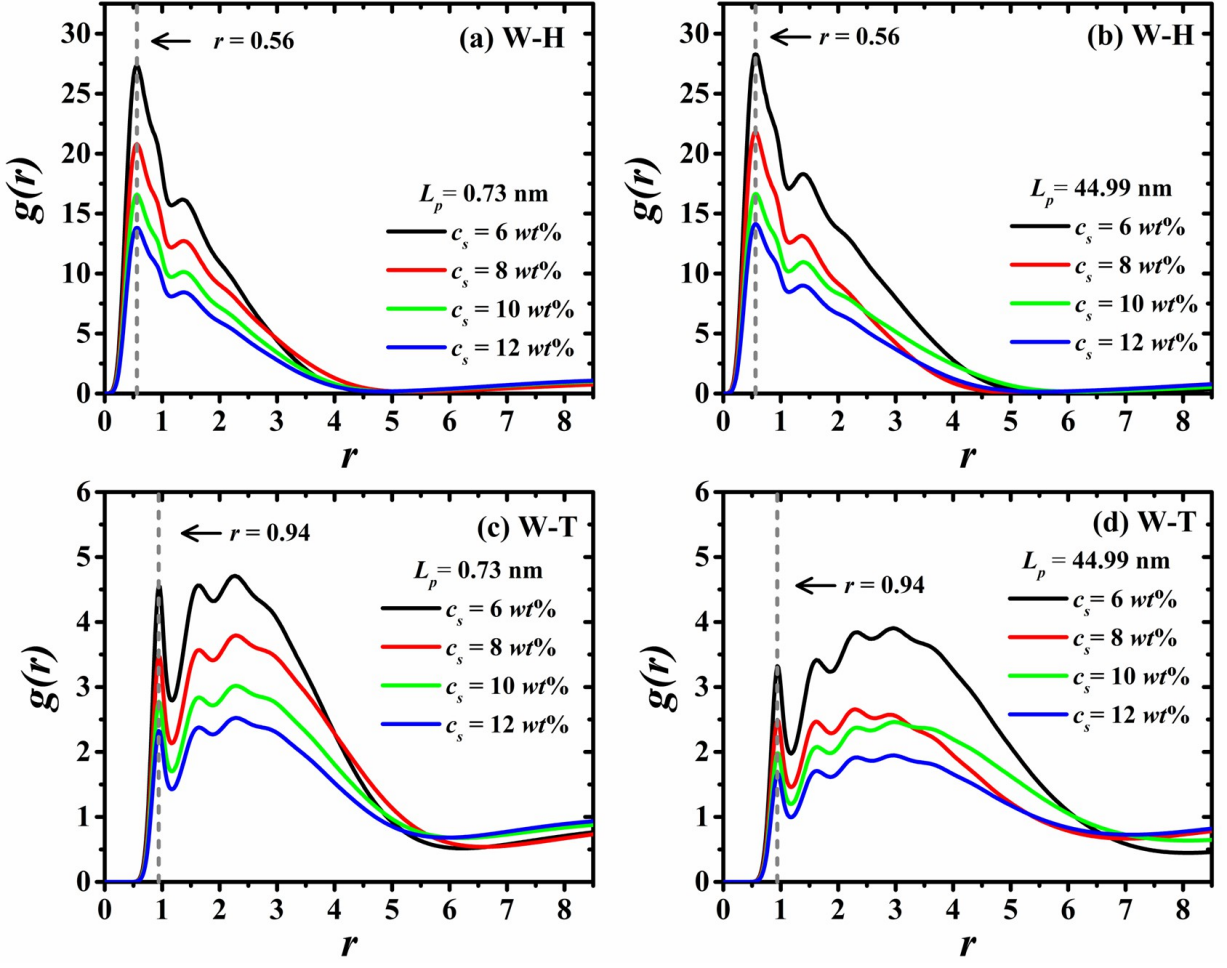
Radial distribution functions, *g(r)*, between water-surfactants’ heads (panels (a) and (b)) and water-surfactants’ tails (panels (c) and (d)) at the four different surfactant concentrations, *c*_*s*_. In the left-hand panels ((a) and (c)) are shown the *g(r)*’s of the systems made of surfactants with the lowest persistence length (*L*_*p*_ = 0.73 nm), whereas the right-hand panels ((b) and (d)) shows the *g(r)*’s of the analogous systems with stiffer surfactants (*L*_*p*_ = 44.99 nm). Vertical dashed lines show the location of the first maximum of the *g(r)* function.

Having shown that the HT5 surfactant model used here leads to the formation of stable RMs, now the analysis of the structural features of the water/HT5 aggregates is presented. This is done through the calculation of the averaged radial distribution functions (RDFs), *g(r)*, for specific pairs of components of the systems. The RDFs allow one to understand the effects produced by *L*_*p*_ and *c*_*s*_ on the formation of RMs and provide information about the local structure of the system. Fig 5 shows the RDFs between the water beads and the individual components of the surfactants, such as the head groups (W-H, Fig 5(A) and 5(B)) and the tail groups (W-T, Fig 5(C) and 5(D)). For clarity, only the RDFs for the most flexible (*L*_*p*_ = 0.73 nm) and the most rigid surfactant models (*L*_*p*_ = 44.99 nm) are shown in Fig 5.

The spatial correlation between water beads and surfactants’ heads, Fig 5(A) and 5(B), show the closest spatial correlation of all RDFs, since these two components make up the core of the aggregates. Although all water beads are trapped inside the RMs nuclei, the (W-H) RDFs show closer spatial correlation than the (W-W) RDFs; see also S3 Fig in the S1 File. This is expected because the repulsive force between the water beads and the surfactants’ head groups is the lowest among all the other pair of components, including those between particles of the same type; see Table 1. The information provided by the (W-T) RDFs, shown in Fig 5(C) and 5(D), confirms that the RMs nuclei are surrounded by the surfactant tail groups. This is because the (W-T) correlation is found to be displaced to larger relative distances, in comparison with the (W-H) correlation. This conclusion is supported by the vertical dashed lines in Fig 5(C) and 5(D), which indicate that the relative position of the first maximum of the (W-T) RDFs (*r* = 0.94), is located at a larger relative distance than the position of the first maximum of the (W-H) RDFs (*r* = 0.56 in Fig 5(A) and 5(B)). These structural characteristics are typically obtained when modeling RMs with linear HT surfactant models, as reported by Mayoral *et al* [2]. who study the water/HT4 self-assembly into RMs in nonpolar solvents.

The influence of surfactant concentration on the RDFs between the water beads and the surfactant head- and tail-groups, Fig 5(A)–5(D), respectively, is analyzed at short and large relative distances. At short distances, the spatial correlation between water beads and HT5 surfactant chains shows that the tail groups surround the RMs nucleus made up of the water beads and head groups. On the other hand, both (W-H) and (W-T) RDFs show weaker correlation as *c*_*s*_ increases, because the average intensity of *g*(*r*) decreases with the increasing concentration of water beads and surfactant chains in the system. Additionally, the RDFs in Fig 5 reveal that the number of aggregates formed in the system affects directly the spatial correlation between water beads and HT5 at relatively large distances. That is because at large distances, the water beads in the RMs nucleus only correlate with the surfactants’ head- or tail-groups belonging to other aggregates. Therefore, the (W-H) and (W-T) correlations tend to have different decaying rates at large relative *r* values, depending on the number of RMs. It is typically found that the (W-H) and (W-T) RDFs tend to decay at relatively short distances if the number of aggregates is higher; see Fig 5(A) and 5(C). It is remarked that these effects occur because in this range of concentrations (6 *wt*% ≤ *c*_*s*_ ≤ 12 *wt*%), the water beads/HT5 aggregates formed are always RMs-like structures, with no free water beads found in the system.

The influence of the surfactant’s persistence length on the structure of the micelle nuclei is minimal at short distances, as seen in the (W-H) RDFs in Fig 5(A) and 5(B). Focusing on large relative distances, say at *r* > 5, the (W-H) correlations only occur between different micelle’s nuclei. Therefore, the (W-H) *g*(*r*)’s tend to unit at large relative distances because in all cases exists more micelles surrounding the (W-H) beads. The consequences of increasing of *L*_*p*_ on the (W-T) spatial correlations are shown in Fig 5(C) and 5(D), corresponding to the most flexible and most rigid surfactants, respectively. Firstly, it is noted that the intensities of the (W-T) *g*(*r*)’s decrease as the surfactants gain rigidity, since for stiffer surfactants the aggregation number grows; see also Fig 4. On the other hand, it is found that the (W-T) spatial correlation prevails over further distances for the stiffer surfactants because the tail groups are more stretched around the RMs nuclei.

**Fig 6:**
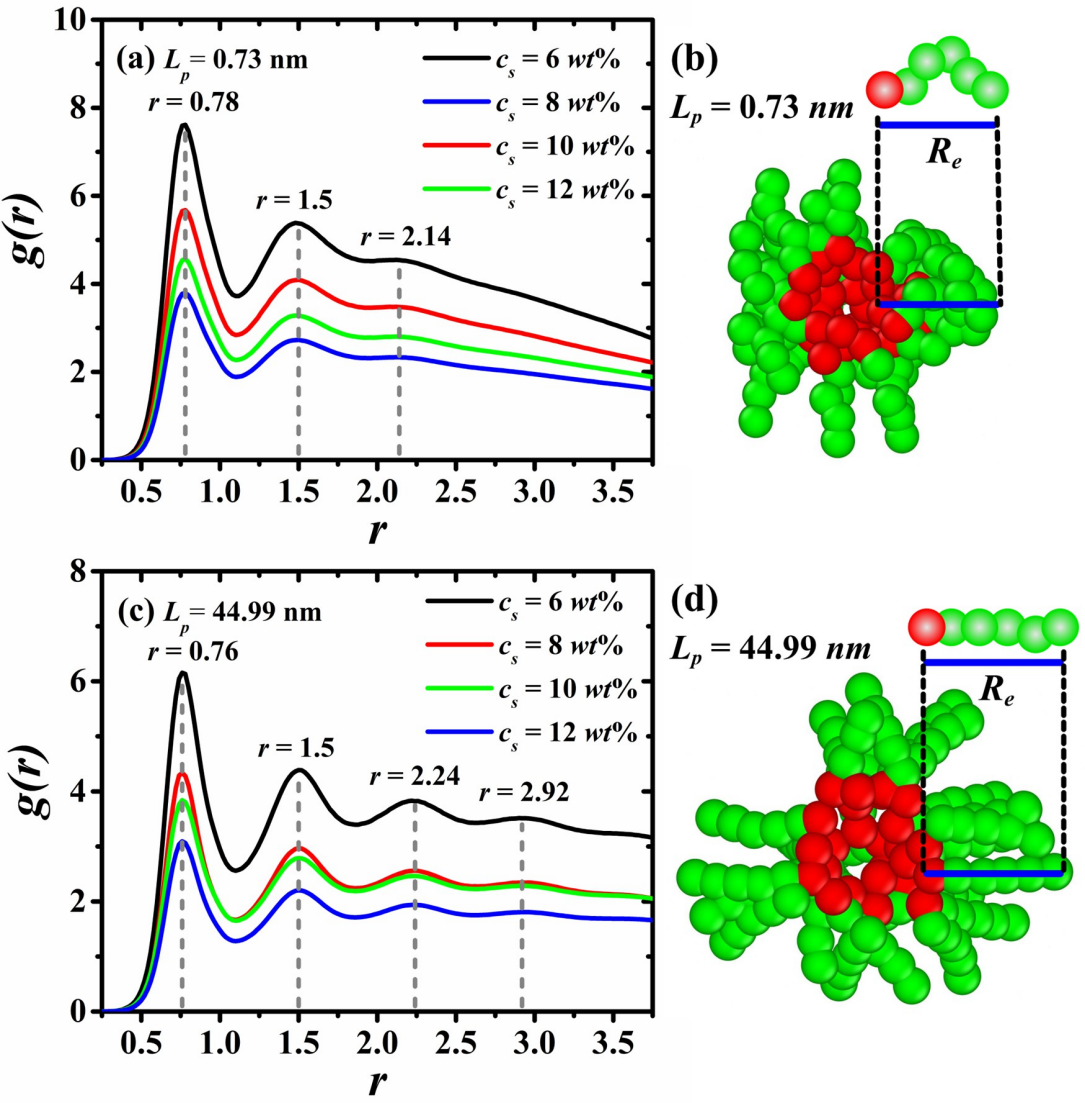
Averaged RDFs between surfactants’ head- and tail-groups, *g(r)*, for the most flexible ((a), *L*_*p*_ = 0.73 nm) and the most rigid ((c), *L*_*p*_ = 44.99 nm) surfactants. Panels (b) and (d) show snapshots of typical RMs, sliced to see their core, where water contained inside the RM were removed to better visualization of the HT5 structure. The vertical dashed lines in panels (a) and (c) indicate the relative position of each maximum. *R*_*e*_ in panels (b) and (d) refers to the average end-to-end distance of the HT5 surfactant in each case (see Eq (7) and Fig 7).

Next, the structure surrounding the RMs nuclei was studied through the analysis of the spatial correlation between the surfactants’ head- and tail-groups. Fig 6 shows the RDFs between the surfactants’ head- and tail-groups (H-T), accompanied by a snapshot of a typical RM for the most flexible (Fig 6(A) and 6(B)) and the most rigid (Fig 6(C) and 6(D)) surfactants. Fig 6 shows the influence of the surfactants’ persistence length on the structure of the beads covering the RMs nuclei, revealing the average structure of the HT5 surfactants in the RMs. These results show that the arrangement of tail beads of the most flexible surfactants enveloping the RMs nuclei is more compact than those of the most rigid surfactants, whose stretched configuration is responsible for the increase in the size of the RMs. The number of maxima observed in the (H-T) RDFs, indicated by the vertical dashed lines in Fig 6(A) and 6(C), confirm the compact structure for the most flexible case. In Fig 6(A), the third maximum is barely noticeable, whereas for the most rigid case, in Fig 6(C), up to a fourth maximum can be seen, indicating a stretched surfactant configuration.

To gain further understanding of the HT5 structure in the RMs, the mean end-to-end distance $\langle R_{e}\rangle$ of HT5 surfactant is calculated, as follows:

$$\langle R_{e}\rangle =\frac{1}{n}\underset{n}{\sum }\langle \left|r_{n}^{l}-r_{n}^{f}\right|\rangle ,$$ (7)

where $r_{n}^{l}$ and $r_{n}^{f}$ are the position vectors of the last (*l*) and first (*f*) particles of the n-th surfactant chain, respectively. The vertical bars, | |, refer to the magnitude of the vector $r_{n}^{l}-r_{n}^{f}$, and the angular brackets represent the average over time. Table 4 shows the mean end-to-end distances of the HT5 surfactants.

**Table 4:** Mean end-to-end distance ($\langle R_{e}\rangle$) of the HT5 surfactants, for all persistence length values and surfactant concentrations modeled here. The values of $\langle R_{e}\rangle$ are in reduced DPD units.

*L*_*p*_ [nm]	6 *wt*%	8 *wt*%	10 *wt*%	12 *wt*%
**0.73**	2.6012 ± 0.0089	2.6111 ± 0.0059	2.6119 ± 0.0073	2.6108 ± 0.0039
**2.03**	3.1647 ± 0.0043	3.1687 ± 0.0073	3.1651 ± 0.0030	3.1638 ± 0.0055
**4.29**	3.4459 ± 0.0021	3.4446 ± 0.0025	3.4462 ± 0.0021	3.4475 ± 0.0015
**8.82**	3.5941 ± 0.0011	3.5943 ± 0.0009	3.5953 ± 0.0008	3.5940 ± 0.0006
**44.99**	3.7394 ± 0.0009	3.7393 ± 0.0006	3.7384 ± 0.0007	3.7390 ± 0.0005

The results listed in Table 4 show that $\langle R_{e}\rangle$ increase as the surfactant persistence length does, tending to their fully extended length (*R*_*FEL*_) as the surfactant stiffness increases. To define the fully extended length of the surfactant, the average bond distance of adjacent surfactant’s beads was used. In this case, such bond distance is found to be $\langle b_{0}\rangle =0.76r_{c}^{*}$, indicated by the first maximum in Fig 6(C). Thus, the fully extended length of the surfactant is defined as:

$$\langle R_{FEL}\rangle =N_{b}\langle b_{0}\rangle$$ (8)

with *N*_*b*_ as the total number of bonds in the surfactant.

**Fig 7:**
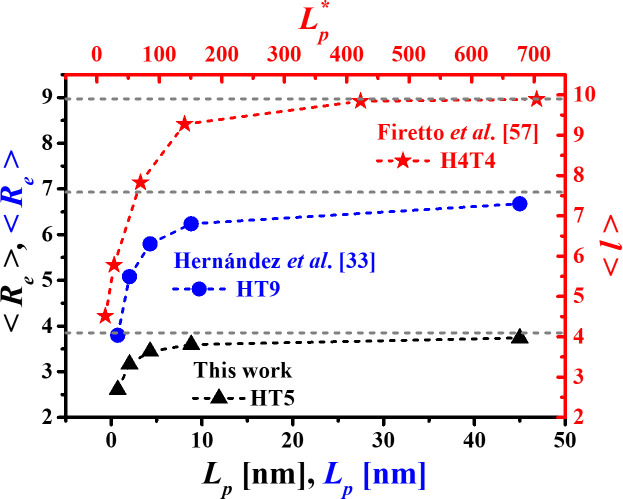
Mean end-to-end distance $\langle R_{e}\rangle$ as a function of the persistence length (*L*_*p*_) obtained from the average over all the surfactant concentrations. Here, the results of this work (black triangles) are compared with those for the HT9 model (blue circles) [33], and with the results of Firetto *et al*. [46] for the H4T4 surfactant model (red stars). The horizontal dashed lines indicate the fully extended length $\langle R_{FEL}\rangle$ predictions for each surfactant model. The vertical and top axes are expressed in reduced units.

The results obtained for $\langle R_{e}\rangle$ as a function of persistence length are shown in Fig 7, where the average of $\langle R_{e}\rangle$ is reported over the four concentration values, since $\langle R_{e}\rangle$ does not show significative differences between the *c*_*s*_ values (see Table 4). In Fig 7, our predictions are compared with those reported by Hernández *et al*. [33] for larger, HT9 surfactant chains. The results of Firetto *et al*. [46], who study the effects of the stiffness on the micellization of the H4T4 surfactant model, are included as well.

Additionally, the dynamics of the RMs are studied by means of their self-diffusion coefficient, *D*, as a function of the surfactants’ persistence length, for each of the surfactant concentrations. The self-diffusion coefficient *D* is calculated from the mean-square displacements (MSD) of the center of mass of the RMs, during the last 10 simulations blocks, which is equivalent to 1 × 10^5^ Δ*t* ≈ 9 ns, through Einstein’s relation [47]

$$MSD=\langle \frac{1}{N}\sum_{i=1}^{N}\;{\left|r_{i}(t)-r_{i}(0)\right|}^{2}\rangle =6Dt.$$ (9)

**Fig 8:**
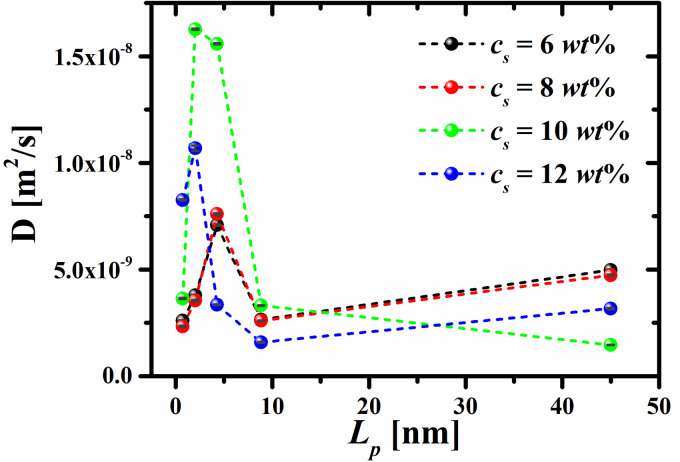
Self-diffusion coefficient (*D*) of the water/HT5 RMs as a function of the surfactant persistence length (*L*_*p*_), for the different surfactant concentrations. The dashed lines are only guides for the eye.

The self-diffusion coefficients of the RMs are shown in Fig 8. The values of *D* reported in this work are comparable to those of RMs used to encapsulate water molecules in experiments [48, 49]. It is also found that the combined influence of the surfactants’ concentration and persistence length can improve the mobility of water beads/HT5 aggregates, in the regime of lower concentration, for flexible surfactants. Our results show that, in this latter regime, the diffusion capacity of the RMs is low because a higher number of aggregates are formed; therefore, the RMs find it more difficult to move freely. However, when the aggregates are assembled by semiflexible HT5 surfactants, their diffusion is enhanced, although the concentration dependence of *D* is nonmonotonic; see Fig 8. Lastly, it is found that the diffusion of the aggregates assembled with HT5 surfactants that are close to their fully extended length is smaller than that of the RMs formed with semiflexible surfactants. This is due to the high steric hindrance of the RMs made up of surfactants with high rigidity.

## 4. Conclusions

In this work, the interplay between surfactant concentration and persistence length on structural and dynamic properties of water/HT5 surfactant aggregates, forming reverse micelles as water-droplet carriers was investigated. It is found that increasing the surfactants’ concentration leads to higher number of water/HT5 aggregates, although this number is generally reduced when the persistence length of the surfactants is increased. The aggregates formed are roughly spherical in shape; however, the structure of the surfactants’ tail groups surrounding the RMs nuclei depends on the surfactants’ rigidity. More flexible surfactants yield more compact aggregates. Our results prove that there is synergy between *c*_*s*_ and *L*_*p*_, leading to enhanced diffusion of the water beads/HT5 aggregates, for certain surfactant concentrations and persistence lengths. The self-diffusion coefficient of the RMs is found to depend on the cooperative behavior between *c*_*s*_ and *L*_*p*_. Also, it is noted that diffusion is improved when the number of RMs is relatively low, to ensure ample motion capacity over the entire system. Additionally, the HT5 surfactant chains must be flexible enough so that the structure enveloping the RMs nuclei is as compact as possible. Otherwise, having numerous micelles in the system leads to low RMs diffusion. On the other hand, having RMs with highly stretched external structures increases the steric hindrance of the aggregates, which in turn reduces their diffusion. These results are expected to be useful in industrial and technological applications where RMs are used. In particular, they should provide insights into processes where the use of RMs and the selection of surfactants to form such aggregates are crucial, such as in drug delivery [50], antibiotic recovery [51], protein recovery [14], and enhanced oil recovery [52].

## Supporting information

S1 File (DOCX)
