# Supplementary material for: Synergy between surfactants’ stiffness and concentration on their self-assembly into reverse micelles as water droplet carriers in nonpolar solvents
Source: PLoS One. 2024 Feb 2;19(2):e0294913. doi: 10.1371/journal.pone.0294913 (PMC10836690; doi:10.1371/journal.pone.0294913)
Supplement: S1 File — (DOCX) [file pone.0294913.s001.docx]

**Supplementary Information**

**for**

**Synergy between surfactants’ stiffness and concentration on their self-assembly into reverse micelles as water droplet carriers in nonpolar solvents**

J. D. Hernández Velázquez and A. Gama Goicochea^[[1]](#footnote-1)^

Tecnológico Nacional de México, Tecnológico de Estudios Superiores de Ecatepec, División de Ingeniería Química y Bioquímica, 55210 Estado de México, Mexico.

**ABSTRACT**

Additional information is provided here in support of the results reported in the main article. Figure S1 contains the supplementary information for Fig. 2 in the main article, showing the density profiles of all systems studied in this work. Figure S2 contains the supplementary results for Fig. 4 in the main article, which show the time evolution of the number of reverse micelles formed during the self-assembly process.

**Fig. S1**. Reduced density profiles of water beads (W, blue lines), surfactants’ head groups (H, red lines), and surfactants’ tailgroups (T, green lines) of the last simulation block for the systems at different surfactant’s persistence length ($L_{p}$) and concentrations ($c_{s}$). Panels (a)-(d) correspond to the systems containing surfactants with a persistence length $L_{p}=0.73 \mathrm{nm}$; panels (e)-(h) correspond to the system with surfactants of $L_{p}=2.03\mathrm{nm}$. Panels (i)-(l) show the results for surfactants of $L_{p}=4.29 \mathrm{nm}$. Panels (m)-(p) show the density profiles in reduced units of the surfactants with $L_{p}=8.82 \mathrm{nm}$, and panels (q)-(t) show the reduced density profiles of the systems with surfactants of $L_{p}=44.99 \mathrm{nm}$. The density profiles of the nonpolar solvent are omitted for clarity. Scales on both axes are reported in reduced DPD units. The results shown in this figure are intended to complement the results of Fig. 2 in the main article.

**Fig. S2**. Time evolution of the number of reverse micelles ($N_{RMs}$) assembled during the last $18.072 \mathrm{ns}$ of each simulation, counting the $N_{RMs}$ every $451.8 \mathrm{ps}$. This figure includes additional results to those of Fig. 4 in the main article.

**Fig. S3**. Radial distribution functions, *g(r)*, between water beads (W-W), for the systems with RMs formed by the most flexible (a) and the most rigid surfactants (b), for all the surfactant concentration ($c_{s}$) studied. The vertical dashed lines refers to the postion of the first maximum of the (W-W) RDFs, which is located at a relative larger distance than the first maximum of the W-H RDFs (see Fig. 5(a)-(b) in the main article).

1. Corresponding author. Email: [agama@alumni.stanford.edu](mailto:agama@alumni.stanford.edu) [↑](#footnote-ref-1)
